# Myocardial involvement in end-stage renal disease patients with anemia as assessed by cardiovascular magnetic resonance native T1 mapping: An observational study

**DOI:** 10.1097/MD.0000000000039724

**Published:** 2024-11-15

**Authors:** Lin Chen, Rong Xu, Huayan Xu, Zhigang Yang, Yi Zhang, Zhenlin Li, Chunchao Xia, Li Rao, Yingkun Guo

**Affiliations:** a Department of Radiology, The Affiliated Suzhou Hospital of Nanjing Medical University, Suzhou Municipal Hospital, Gusu School, Nanjing Medical University, Suzhou, Jinagsu, China; b Department of Radiology, West China Second University Hospital, Sichuan University, Key Laboratory of Obstetric & Gynecologic and Pediatric Diseases and Birth Defects of Ministry of Education, Chengdu, Sichuan, China; c Department of Radiology, West China Hospital, Sichuan University, Chengdu, Sichuan, China; d Department of Radiology, National Key Laboratory of Biotherapy, West China Hospital, Sichuan University, Chengdu, Sichuan; e Department of Cardiology, West China Hospital, Sichuan University, Chengdu, China.

**Keywords:** anemia, cardiovascular magnetic resonance, fibrosis, T1 mapping

## Abstract

Cardiovascular disease has become to the main cause of death in the patients with end-stage renal disease (ESRD), and anemia is associated with increased cardiovascular morbidity and mortality in these patients. This study aimed to explore the impact of anemia on myocardial fibrosis using T1 mapping technique in patients with ESRD. A total of 128 subjects including 98 ESRD patients (65 with anemia, 33 without anemia) and 30 normal controls were enrolled. All subjects were underwent cardiovascular magnetic resonance to obtain cardiac cine and T1 mapping images. As potential markers of fibrosis, native T1 values and global longitudinal strain derived by feature-tracking technique were compared. Differences between 3 groups were analyzed using one-way analysis of variance. Associations between variables were assessed by Pearson and Spearman correlation coefficient appropriately. An independent association was identified by the multiple stepwise linear regression analysis. Intraclass correlation was applied to assess observer variability. In all ESRD patients, native T1 values were significantly longer than those of normal controls (global T1, 1357 ± 42 ms vs 1275 ± 48 ms, *P* < .001). Global T1 value in ESRD patients with anemia was significantly higher (1375 ± 36 ms) compared to that in ESRD patients without anemia (1322 ± 25 ms) and normal controls (1275 ± 48 ms), respectively (all *P* < .001). Global T1 correlated with hemoglobin negatively (R= −0.499, *P* < .001). Multiple stepwise linear regression analysis presented the anemia is independently associated with global T1 (*R* = 0.607, *P* < .001). Global longitudinal strain was remarkably reduced in ESRD patients with anemia in comparison to those without anemia (*P* < .001). Diffuse myocardial fibrosis could be detected by native T1 mapping in ESRD patients with long-term anemia. Anemia is an important factor in myocardial fibrosis in ESRD patients, and the evaluation of myocardial involvement is worth considering for clinical management.

## 1. Introduction

Cardiovascular disease has become to the main cause of death in the end-stage renal disease (ESRD) patients. Cardiovascular morbidity and mortality in ESRD patients is about 10 to 20 times higher than in the general population.^[1–4]^ Although increased cardiac mortality has been considered as a consequence of nonatherosclerotic processes, sudden cardiac death is a significant characteristic in ESRD patients.^[5,6]^ Myocardial fibrosis is likely to contribute to the ultimate development of cardiac death.

Anemia affects 60%–80% of patients with renal impairment,^[7,8]^ and it is related to elevated cardiovascular morbidity and mortality in ESRD patients.^[9]^ Iron deficiency has become an important cause of anemia since the use of recombinant human erythropoietin became common in ESRD patients.^[10–12]^ Anemia results in reduction of oxygen supply to the tissues which then leads to compensatory changes that increase the cardiac output.^[13]^ Anemia causes a series of changes in cardiac structure and function, such as myocardial ischemia, myocardial hypertrophy, cardiac dilatation, arrhythmia, heart failure, and even cardiac death.^[14,15]^ Given the sampling error with myocardial biopsy and the risk of serious complications (0.6%–0.8%),^[16]^ a noninvasive method of quantitatively assessing myocardial tissue characterization is required.

Cardiovascular magnetic resonance (CMR) is a useful tool for the detection of cardiac tissue characteristic. T1 relaxation time describes the exponential recovery of the longitudinal component of magnetization back towards its thermal equilibrium. Native T1 value, without the need for gadolinium contrasts, is considered as a viable alternative to late gadolinium enhancement (LGE) and it has been revealed to associate with myocardial fibrosis which has been confirmed by histological examination.^[17,18]^ Furthermore, increased native T1 values have been demonstrated in conditions correlated with myocardial fibrosis in some myocardial diseases.^[17,19,20]^

Myocardial fibrosis has been demonstrated in the ESRD population by contrast-enhanced CMR imaging.^[21,22]^ Rutherford et al have shown that increased T1 values in ESRD population may be representative of the myocardial fibrosis.^[23,24]^ In another study, Shah et al have similarly shown that native T1 values are significantly higher in the ESRD population when compared to normal cohorts.^[25]^ However, the evaluation of the impact of anemia on myocardial fibrosis (and therefore on native T1 values) has not been performed yet.

## 2. Objectives

The aims of this study were to explore the potential impact of anemia on myocardial fibrosis using T1 mapping in ESRD patients and to investigate whether hemoglobin levels could be independently related to native T1 values. We hypothesized that the presence of anemia will be associated with myocardial fibrosis, and T1 values would be higher in the ESRD patients with anemia compared to those without anemia.

## 3. Methods

### 3.1. Study population

All of 98 ESRD patients were recruited from the nephrology department. Patients were diagnosed based on the previously established criteria in the guideline.^[26]^ All patients were on regular dialysis (twice a week) and in routine oral iron therapy (polysaccharide iron complex, 600 g/d). Briefly, patients were included if the eGFR was <15 mL/min/1.73 m^2^, they were ≥18 years old, and there was no preexist anemia. Patients were excluded if they had contraindications to CMR, severe arrhythmia such as atrial fibrillation, known hypertrophic cardiomyopathy or dilated cardiomyopathy. Of the 108 patients who met the above criteria, 10 patients were excluded from the study due to death (2 patients), artificial joint implantation (1 patient), pacemaker implantation (1 patient), severe arrhythmia (3 patients), and patient refusal to undergo CMR scanning (3 patients). In total, 98 patients were enrolled into 2 groups: 33 patients without anemia (hemoglobin level ≥ 130 g/L in males, ≥120 g/L in females) and 65 patients with anemia (hemoglobin level < 130 g/L in males, <120 g/L in females) according to the guideline.^[27]^ In these patients, 30.3% of patients without anemia and 41.5% of patients with anemia were males. 30 healthy volunteers were included into the control group, without prior cardiac history or known cardiac risk factors, not on any cardiovascular medications and with normal electrocardiograms. Volunteers were excluded if they were younger than 18 years old and had any renal disease or anemia. This study has been approved by the local ethics committee, and informed consent was acquired from all participators.

### 3.2. Cardiac magnetic resonance

CMR was performed in a 3T MRI scanner (Skyra, Siemens Medical Solutions, Erlangen, Germany) using an 18-channel body phased-array coil. In the ESRD groups, CMR was performed on a post-dialysis day. All the sequences were obtained within an end-expiration phase with breath-holding and electrocardiographically gated. The scan protocol included cine imaging with steady-state free precession and T1 mapping sequence. The short-axis cine images and long-axis cine images in 2, 3, and 4 chamber were obtained (repetition time: 41.6 ms; flip angle: 40°; echo time: 1.51 ms; pixel matrix: 254 × 176; voxel size: 1.5 × 1.3 × 8 mm^3^; slice thickness: 8 mm; bandwidth: 965 Hz/pixel). A series of basal, middle, and apical T1 maps were acquired in short-axis slices (repetition time: 278.56 ms; flip angle: 35°; echo time: 1.12 ms; pixel matrix: 136 × 215; voxel size: 1.4 × 1.4 × 8 mm^3^; slice thickness: 8 mm; bandwidth: 937 Hz/pixel) using a motion-corrected, modified Look-Locker inversion recovery sequence by 5 (3) 3 acquisition pattern without contrast administration and were used to generate the T1 map and values.

### 3.3. Image analysis

Analysis of all CMR images was performed offline using commercial software (cvi42, Circle Cardiovascular Imaging, Inc.). In the serial short-axis slices, the endocardial and epicardial borders were manually outlined by 2 experienced observers (with 3 years of experience in CMR) at the end-diastolic and end-systolic phases. Analysis of the left ventricular (LV) volumes and myocardial deformation parameters, including LV ejection fraction (LVEF), LV end-systolic volume, LV end-diastolic volume, LV stroke volume, LV mass, and global longitudinal strain (GLS), were performed according to the standard methods.^[28]^ GLS was derived from all 3 long-axis cine-views using the feature-tracking technique. LV systolic dysfunction was defined as LVEF < 55%.^[29]^ The segment of T1 maps was divided in line with the American Heart Association 16-segment model.^[30]^ The regions of interest (ROIs) in T1 maps were defined by user semiautomated and it should be standardized conservatively for similar size and shape, particular carefully delineate ROIs with appropriate separation of border from myocardial tissue boundary.

### 3.4. Statistical analysis

Analysis of data was operated on SPSS software (version 19.0; Chicago, IL). Mean ± standard deviation (SD) and median (interquartile ratio, IQR) according to the baseline distribution are the patterns for all data to be presented as. Categorical data are presented as numbers and percentages. One-way analysis of variance with post hoc Bonferroni correction were performed to compare the baseline characteristics and CMR parameters between ESRD patients with or without anemia, and normal controls. Pearson and Spearman correlation coefficients were used appropriately to assess the correlations within different variables. Multiple stepwise linear regression analysis was performed to evaluate those with an independent association, into which variables with a remarkable association with T1 value in correlation analysis would be entered. The intraclass correlation coefficient was used to assess the inter- and intraobserver variabilities of T1 measurements in 15 randomized-selected patients. Two-tailed *P* values of <.05 were considered statistically significant difference.

## 4. Results

### 4.1. Baseline

Baseline demographic characteristics and the laboratory examination results of the groups are shown in Tables 1 and 2. There were no significant differences in sex, age, heart rate, or blood pressure between ESRD with and without anemia. 25 participants (38.4%) in ESRD with anemia had left ventricular systolic dysfunction, as well as 9 participants (27.2%) in ESRD without anemia. ESRD patients without anemia had higher hemoglobin and serum ferritin level compared to those with anemia. LVEF in ESRD patients with or without anemia was lower than in normal controls. Within these groups, they have similar LV mass. Other clinical indicators and cardiac parameters of these groups are presented in Table 2.

**Table 1:** Baseline demographic characteristics and clinical data of ESRD patients.

Variable	ESRD without anemia (n = 33)	ESRD with anemia (n = 65)	*P* value
Dialysis modality [%]			
Hemodialysis	90.9 [30]	90.7 [59]	.98
Peritoneal dialysis	9.1 [3]	9.3 [6]	.98
Duration of dialysis (yr)	3.5 (2–6)	3 (1.5−5.5)	.86
Age at initiation of dialysis (yr)	52 ± 13	54 ± 16	.58
History of kidney transplantation [%]	3 [1]	1.5 [1]	.62
Diabetes [%]	6.1 [2]	7.7 [5]	.98
Hypertension [%]	78.8 [26]	78.5 [51]	.97
Myocardial infarction [%]	3 [1]	6.2 [4]	.85
Ischemic heart disease [%]	6.1 [2]	10.8 [7]	.69
Dyslipidaemia [%]	0 [0]	3.1 [2]	.55
Peripheral vascular disease [%]	24.2 [8]	21.5 [14]	.76
Hb (g/L)	128 ± 8	88 ± 19	<.001
SF (ng/mL)	548.3 (148.6–1448)	159.1 (68.2−606.5)	.01
Total cholesterol (mmol/L)	4.2 ± 0.9	3.7 ± 0.8	.01
LDL cholesterol (mmol/L)	2.1 ± 0.7	1.9 ± 0.6	.14
Cr (μmoI/L)	828 (605−1037)	761 (580−1058)	.39
Glu (mmol/L)	5.6 ± 2.5	6.1 ± 2.9	.45
SBP (mm Hg)	141 ± 26	139 ± 23	.67
DBP (mm Hg)	87 ± 17	84 ± 14	.33

Cr = creatinine, DBP = diastolic blood pressure, ESRD = end-stage renal disease, Glu = glucose, Hb = hemoglobin, LDL = low density lipoprotein, SBP = systolic blood pressure, SF = serum ferritin. All data were showed as mean ± standard deviation, median (interquartile range), or percentage [number of participants] appropriately.

**Table 2:** Baseline characteristics and cardiac parameters of the study population.

Variable	Normal controls (n = 30)	ESRD without anemia (n = 33)	ESRD with anemia (n = 65)	*P* value
Age (years)	52 ± 10	56 ± 13*	57 ± 16*	.21
Male [%]	36.7 [11]	30.3 [10]	41.5 [27]	.55
Weight (kg)	75.3 ± 10.3	72.8 ± 11.2	72.4 ± 11.4	.63
BMI (kg/m2)	25.6 ± 2.7	24.5 ± 3.6	24.8 ± 4.2	.81
HR (bpm)	78 ± 6	82 ± 15	79 ± 12	.76
LVESV (mL)	56.7 ± 21.3	53.3 ± 23.4	58.7 ± 22.1	.78
LVEDV (mL)	133.7 ± 33.1	127.1 ± 54.8*	138.7 ± 50.6	.47
LVSV (mL)	71.8 ± 32.6	62.4 ± 21.5	70.1 ± 19.9	.19
LVEF (%)	68.8 ± 6.4	53.6 ± 15.1*	53.5 ± 11.9*	<.001
LVM (g)	84.5 ± 33.3	85.7 ± 31.8	88.3 ± 28.7	.32
CO (L/min)	4.8 ± 1.5	5.0 ± 1.7	5.5 ± 1.8*	.08
Global T1 (ms)	1275 ± 48	1322 ± 25*	1375 ± 36*,†	<.001
Basal T1 (ms)	1251 ± 47	1301 ± 26*	1350 ± 35*,†	<.001
Medial T1 (ms)	1272 ± 58	1319 ± 36*	1370 ± 35*,†	<.001
Apical T1 (ms)	1301 ± 54	1346 ± 32*	1406 ± 50*,†	<.001
GLS (%)	−18.1 ± 1.2	−15.1 ± 3.2*	−13.1 ± 3.3*,†	<.001

ESRD = end-stage renal disease; BMI, body mass index; HR, heart rate, GLS = global longitudinal strain, LVEDV = left ventricular end-diastolic volume, LVEF = left ventricular ejection fraction, LVESV = left ventricular end-systolic volume, LVM = left ventricular mass; CO, cardiac output, LVSV = left ventricular stroke volume. All data were showed as mean ± standard deviation or percentage [number of participants] appropriately. One-way analysis of variance was performed for continuous variables with normal distribution, nonparametric test for continuous variables with non-normal distribution, and chi-squared test for counting variables.

* *P* < .05 vs normal controls.

† *P* < .05 vs ESRD without anemia.

### 4.2. Impact of anemia on myocardial native T1 values

**Figure 1.**
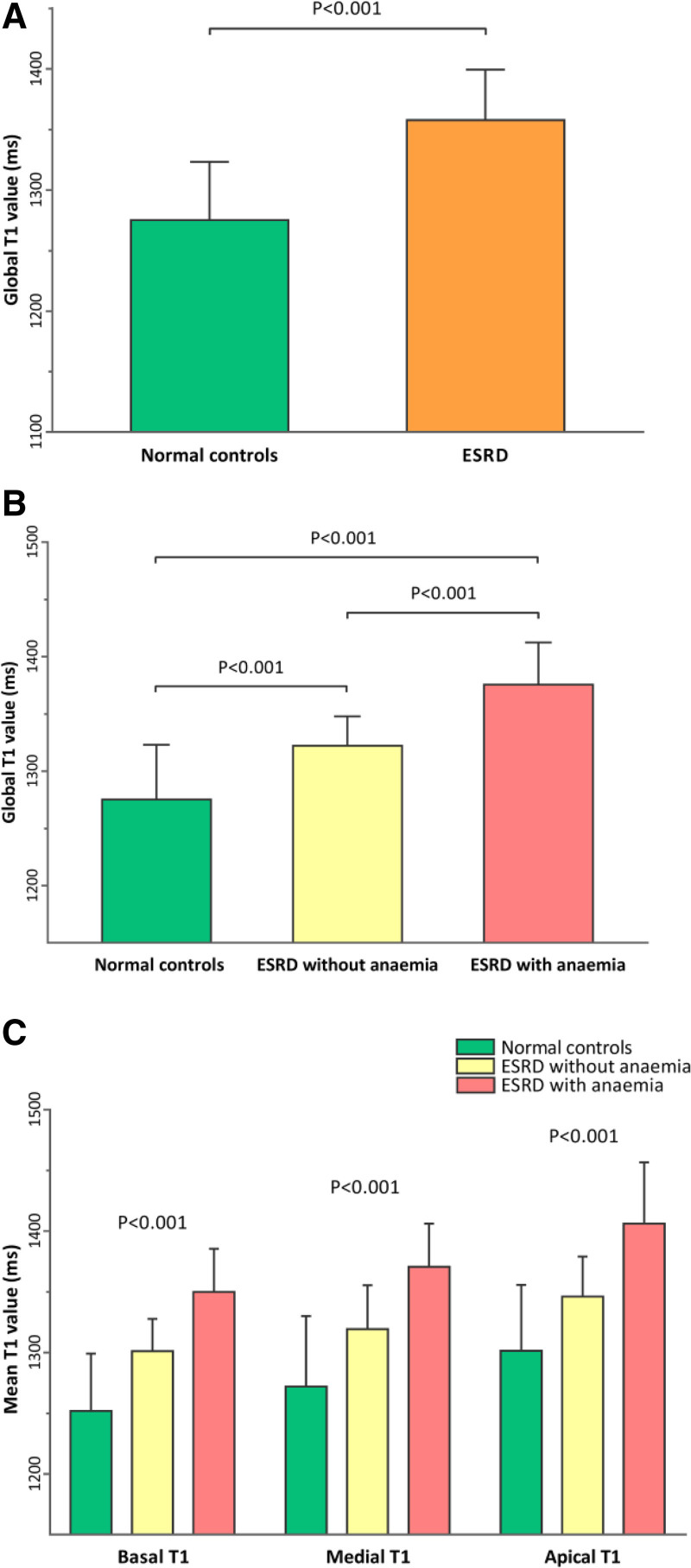
Bar graphs comparing native T1 values in normal controls and ESRD patients. (A) Higher mean global T1 value was seen in patients with ESRD compared to normal controls. (B) Higher mean global T1 value was seen in ESRD patients with anemia compared to ESRD patients without anemia or normal controls. (C) Mean T1 values were significantly longer in ESRD patients with anemia compared to ESRD patients without anemia or normal controls, in all 3 slices (basal, mid, and apical) of the left ventricle.

**Figure 2.**
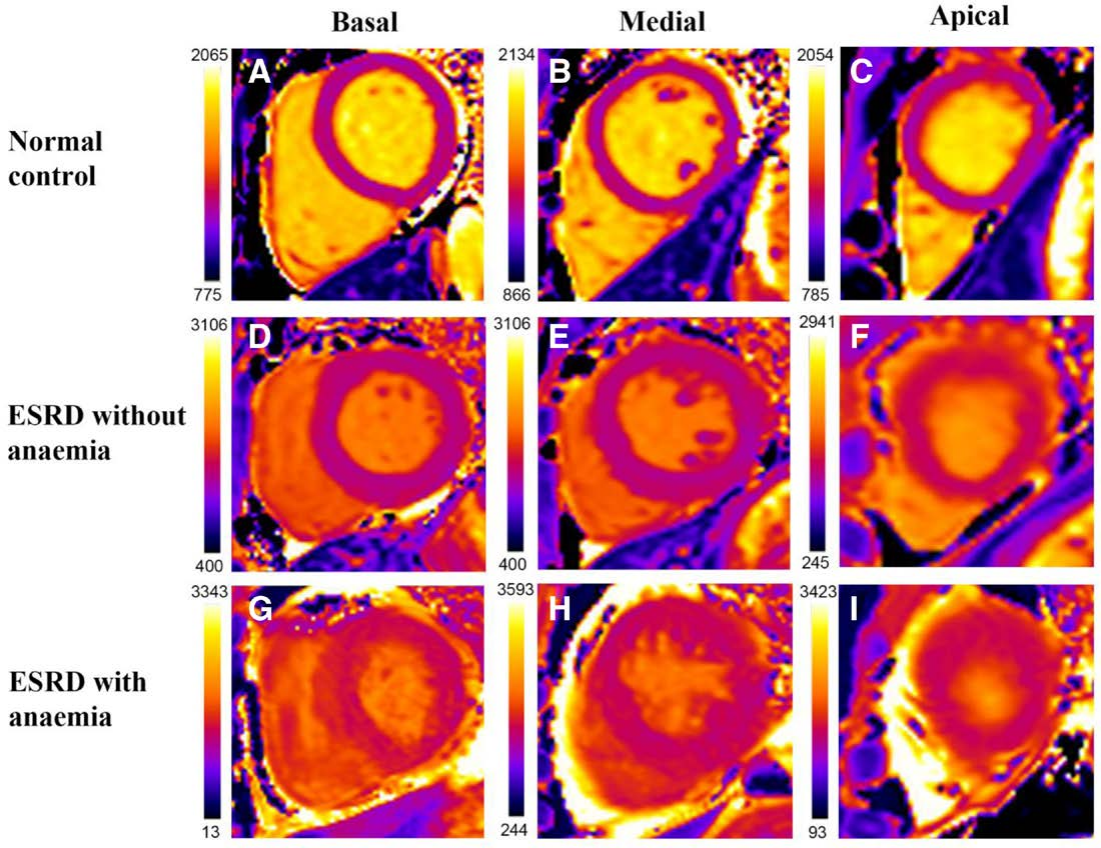
Representative native T1 maps of the basal (A, D, G), medial (B, E, H), and apical (C, F, I) short-axis slices. Typical native T1 maps of 3 participants are presented. Visually, the ESRD patient with anemia had the higher T1 values than the ESRD patient without anemia and healthy volunteer.

Normal volunteers had lower global T1 values (1275 ± 48 ms vs 1357 ± 42 ms, *P* < .001) and higher GLS (−18.1 ± 1.2% vs −13.8 ± 3.4%, *P* < .001) compared to ESRD participants (Fig. 1A). Among the ESRD patients, those without anemia had lower global T1 values (1322 ± 25 ms vs 1375 ± 36 ms, *P* < .001) and higher GLS (−15.1 ± 3.2% vs −13.1 ± 3.3%, *P* < .001) compared to ESRD patients with anemia (Fig. 1B). The above pattern of stepwise change in global native T1 values was also seen in the regional T1 values when the 3 left ventricular slices (basal, mid, and apical) were analyzed separately (Fig. 1C). Moreover, typical T1 maps are shown in Figure 2.

### 4.3. Correlations between CMR parameters and clinical parameters

**Figure 3.**
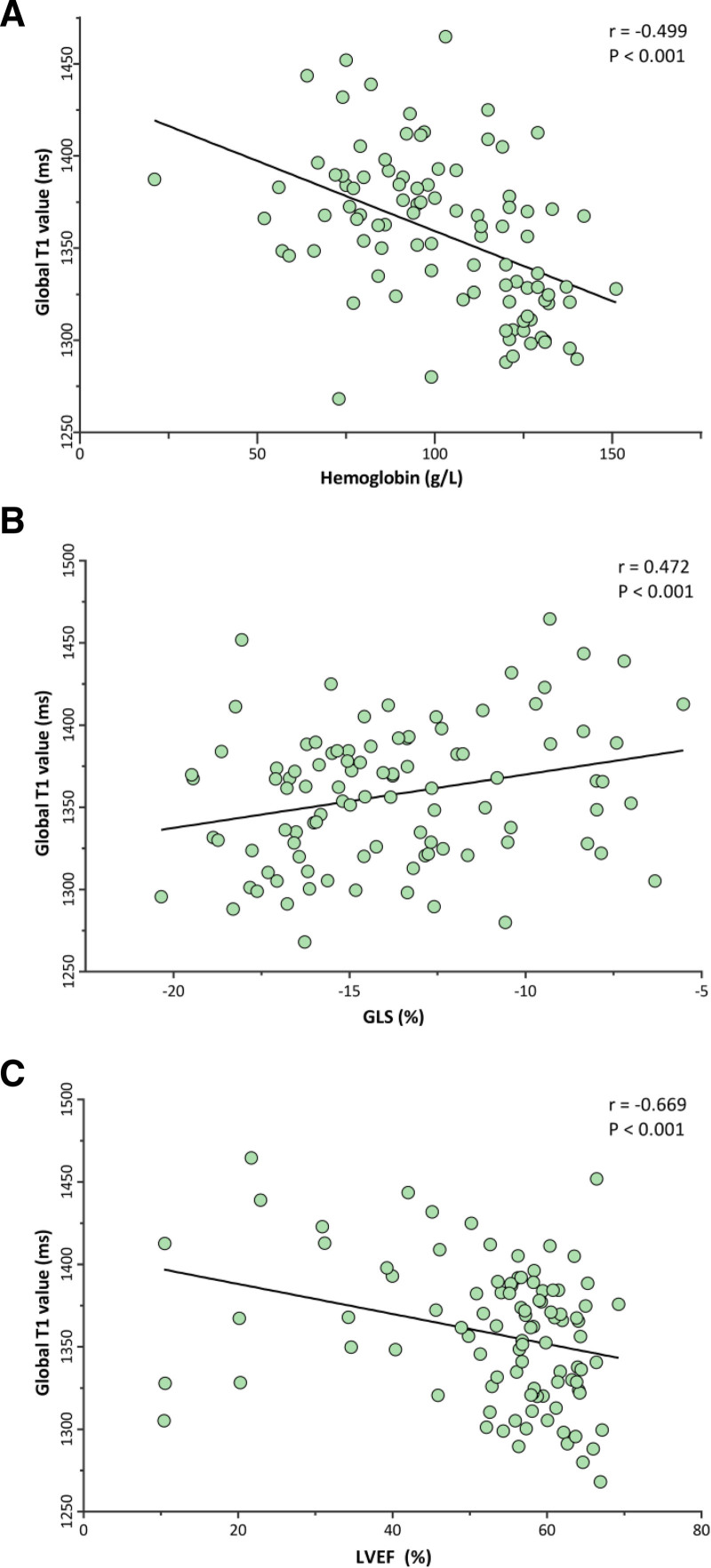
Scatterplots of global T1 value against hemoglobin, GLS, and LVEF in ESRD patients. (A) Relationship between hemoglobin and global T1 value. (B) A positive relationship between GLS and global T1 value. (C) Relationship between LVEF and global T1 value.

In ESRD patients, there was moderate negative correlations seen between global T1 values and hemoglobin, LVEF, and serum ferritin (Table 3 and Fig. 3A). There was moderate positive correlations seen between global T1 values and anemia and GLS (global T1: *R* = 0.472; basal T1: *R* = 0.497; medial T1: *R* = 0.459; apical T1: *R* = 0.445; all *P* < .001) (Table 3 and Fig. 3B). Multiple stepwise linear regression analysis showed that anemia was independently associated with global T1 (*R* = 0.607, *P* < .001), as well as LVEF (*R* = 0.669, *P* < .001) (Fig. 3C). More details are presented in Table 4.

**Table 3:** Univariate analysis of associations between CMR and clinical parameters.

	Global T1		Basal T1		Medial T1		Apical T1	
	*R* value	*P* value	*R* value	*P* value	*R* value	*P* value	*R* value	*P* value
Hb	−0.499	<.001	−0.514	<.001	−0.479	<0.001	−.408	<.001
Anemia	0.629	<.001	0.632	<.001	0.564	<0.001	.549	<.001
SF	−0.227	.025	−0.227	.025	−0.280	0.005	−.288	.004
LVEF	−0.299	.003	−0.260	.01	−0.348	0.002	−.235	.02
GLS	0.472	<.001	0.497	<.001	0.459	<0.001	.445	<.001

GLS = global longitudinal strain, Hb = hemoglobin, LVEF = left ventricular ejection fraction, SF = serum ferritin. All variables were included to perform bivariate correlation analysis using Pearson or Spearman method as appropriate. T1 values showed good correlations with anemia and moderate correlations with Hb or GLS, while they had weak negative correlations with SF.

**Table 4:** Multivariate analysis of factors associated with T1 and GLS.

	Global T1		Basal T1		Medial T1		Apical T1		GLS	
	*R* value	*P* value	*R* value	*P* value	*R* value	*P* value	*R* value	*P* value	*R* value	*P* value
Anemia	0.607	<.001	0.579	<.001	0.564	<.001	0.534	<.001	0.510	<.001
LVEF	0.669	<.001	0.659	<.001	0.642	<.001	0.571	<.001	0.542	<.001

GLS = global longitudinal strain, LVEF = left ventricular ejection fraction. Univariable factors related to T1 values and GLS at *P* < .05 were entered into a multivariable stepwise linear regression analysis, including age, Hb, LDL cholesterol, total cholesterol, anemia, SF, diabetes, hypertension, LVEF, LVESV, and LVEDV.

### 4.4. Reproducibility

Interobserver and intraobserver variability for T1 measurements was acceptable. The intraclass correlation coefficients are presented in Table 5, as well as the average differences within T1 values measured by observers, and the rate of absolute differences to the average values in 4 calculations.

**Table 5:** Observer variability for T1.

	Intraobserver			
	Ratio of absolute Difference to mean (95% CI)	Mean difference (95% CI)	*P* value	ICC (95% CI)
Global T1	0.036 (0.021–0.052)	0.287 (−0.326 to 0.900)	.332	0.95 (0.86–0.98)
Basal T1	0.042 (0.020–0.062)	0.293 (−0.326 to 0.900)	.315	0.94 (0.83–0.98)
Medial T1	0.050 (0.019–0.080)	−0.415 (−1.779 to 0.950)	.525	0.93 (0.80–0.98)
Apical T1	0.057 (0.018–0.095)	−0.236 (−1.224 to 0.784)	.417	0.94 (0.83–0.98)
	Interobserver			
	Ratio of absolute Difference to mean (95% CI)	Mean difference (95% CI)	*P* value	ICC (95% CI)
Global T1	0.028 (0.014–0.043)	−0.270 (−0.802 to 0.262)	.294	0.95 (0.86–0.98)
Basal T1	0.019 (0.011–0.028)	−0.180 (−0.612 to 0.062)	.352	0.96 (0.88–0.98)
Medial T1	0.075 (0.040–0.110)	−1.871 (−3.415 to − 0.328)	.021	0.91 (0.74–0.97)
Apical T1	0.026 (0.016–0.036)	−0.254 (−0.684 to 0.169)	.324	0.95 (0.86–0.98)

CI = confidence interval, ICC = intraclass correlation coefficient.

## 5. Discussion

### 5.1. The main findings

As far as we know, it is the first time to compare native T1 value between ESRD patients with anemia and those without anemia, and we demonstrated that native T1 values are significantly elevated in ESRD patients with anemia. Increased T1 values have previously been considered to be representative of a diffuse myocardial fibrotic process verified by cardiac tissue biopsy in several populations.^[17,23,31]^ In addition, we confirmed that increased native T1 values of ESRD populations could be found in comparison with healthy volunteers. T1 mapping could be an innovative technique to quantitatively evaluate myocardial involvement in ESRD patients with anemia.

### 5.2. Rationale for global T1 analysis

Myocardial fibrosis has been shown to play a role in the pathological process of many diseases which affected cardiac health, and its existence implies a poor prognosis.^[17,32,33]^ It is feasible to confirm the presence of myocardial fibrosis in the majority of populations using LGE technique of CMR. Given that it is contraindicated to make use of gadolinium contrasts in ESRD patients due to the risk of a potentially lethal condition known as nephrogenic systemic fibrosis (NSF), using LGE to confirm the presence of myocardial fibrosis is impracticable. Moreover, LGE is limited in the qualitative detection of diffuse myocardial fibrosis.^[31,34]^ Native T1 could quantitatively detect diffuse myocardial fibrosis without gadolinium contrast use and therefore avoiding the risk of NSF.^[35,36]^ In addition, much evidence supports the notion that T1 mapping indices are influenced by the presence of myocardial edema,^[37,38]^ another putative factor possibly influencing native T1 assessment is volume overload, although contradictory data are currently available in the literature.^[39–41]^ Given the aforementioned confounding factors, T2 mapping could be incorporated into the comprehensive assessment to offset partial interference.

Native T1 mapping, especially in diffuse diseases such as uremic cardiomyopathy, should be assessed with a conservative ROI drawn at mid-septum level in accordance with SCMR consensus documents.^[42,43]^ With the purpose of ensuring that we did not make false assumption that regional higher T1 values represented the presence of myocardial fibrosis, hence the measurement of T1 values was performed including 3 short-axis slices (basal, medial, and apical myocardial of LV). we measured T1 mean values from all 16 ROIs based on the suggested model by American Heart Association.

### 5.3. Impact of ischemic heart disease and diabetes on T1 values

Regionally increased native T1 values could be found in the region of myocardial involvement such as chronic myocardial infarction (CMI).^[44]^ In this study, as the ESRD patients were unscreened, some patients had known traditional ischemic heart disease (IHD). Previously with no concern about NSF which was related to the use of gadolinium contrasts in ESRD population, 2 modes of myocardial fibrosis could been found in ESRD patients, including subendocardial myocardial fibrosis of IHD and diffuse myocardial fibrosis of cardiomyopathy.^[22]^ However, we did not consider the increased T1 values of ESRD patients as a result of traditional IHD. Five ESRD patients (4 with anemia and 1 without anemia) were included with previous history of CMI. Additionally, if we totally eliminated those 5 patients, in fact the mean global, basal, medial, and apical T1 values of ESRD patients had not been altered (global T1 excluding CMI patients 1356 ± 42 ms vs 1357 ± 42 ms including CMI patients), as well as ESRD group without anemia (1321 ± 25 ms vs 1322 ± 27 ms) and ESRD group with anemia (1374 ± 36 ms vs 1375 ± 36 ms).

In our study, 7 ESRD participants (2 without anemia and 5 with anemia) had a history of diabetes. However, the proportion of diabetic patient in ESRD patients with anemia was similar to that in those without anemia. In addition, we have included diabetes to a multivariable stepwise linear regression analysis. So we do not believe that the presence of diabetes has a real influence on the increased T1 values in the ESRD group with anemia.

### 5.4. LVEF, anemia, hemoglobin, serum ferritin, and T1 values

A negative association have been found among LVEF and T1 values. This correlation suggests that as left ventricular systolic dysfunction develops severely, the potential myocardial involvement that result in elevated T1 values evolves simultaneously. In this study, only a few patients had reduced LVEF but most of patients presented with increased native T1 values. These results suggest that native T1 may be a more sensitive marker of myocardial injury in the ESRD population than LVEF. The comprehensive evaluation of cardiac function and myocardial tissue characteristic may have an important role in guiding cardiac intervention in ESRD patients with anemia.

Similarly, we have found that T1 correlates with decreased hemoglobin and serum ferritin and independently correlates with anemia. Hemoglobin often reflects the severity of anemia, which reminds us that anemia may have some impact on myocardial tissue. Iron deficiency, irrespective of the presence of anemia, is considered as a threat to increased death rate for ESRD patients,^[45,46]^ and it could cause myocardial tissue abnormalities due to tissue hypoxia.^[47]^ The above findings suggest that simple tests like hemoglobin and serum ferritin may indicate the severity of myocardial fibrosis in ESRD patients. However, this needs to be confirmed with larger studies.

As reviewed in previous research, in patients with anemia, the status of iron deficiency in serum might be representative of decreased iron-load in myocardial tissue, which could be detected by measuring up-regulation of the myocardial transferrin receptor expression.^[48]^ Interestingly, as the magnetic resonance signal could be affected by paramagnetic effect of myocardial iron, T1 values are characteristically negatively correlated with the concentration of iron.^[49]^ Left ventricular stiffness correlates with peak oxygen uptake,^[50]^ and myocardial stiffness may increase in patients with anemia for reduced peak oxygen uptake. Furthermore, Ellims et al demonstrated a association of mechanism theory between myocardial stiffness and cardiac fibrosis by T1 mapping technique.^[51]^ Importantly, the presence of anemia may play a role in the evolving of myocardial fibrosis in ESRD patients. T1 mapping may become an ideal tool to quantitatively evaluate the myocardial abnormality in ESRD patients with anemia.

### 5.5. Utility of CMR imaging in the ESRD population

The interplay between ESRD, anemia, GLS, myocardial fibrosis and adverse outcomes is interesting. Anemia results in reduced myocardial oxygenation. Blood oxygen level CMR imaging or oxygen-sensitive CMR (OS-CMR) imaging is very sensitive to myocardial tissue oxygenation.^[52]^ It has been shown that there is significantly blunted myocardial oxygenation response, as assessed by stress OS-CMR, to stimulation in ESRD population without history of heart diseases in comparison with healthy controls and patients with hypertension.^[53]^ The major reason for this would be the presence of microvascular coronary artery disease in this group of patients. However, it may be possible that anemia (seen in ESRD patients) could contribute to this response too. Egred et al have shown that scarred myocardium has lower stress OS-CMR response.^[54]^ GLS is a parameter that is thought to mainly relate to replacement fibrosis and/or interstitial fibrosis. Therefore, myocardial fibrosis may result in both impaired GLS and reduced OS-CMR response to stress. It is well known that myocardial fibrosis is associated with adverse cardiovascular outcomes. Similarly, it has been shown the impaired OS-CMR response in ESRD patients may also have prognostic value in its capacity to predict ESRD patients at higher risk of death, nonfatal myocardial infarction, heart failure and ventricular arrhythmias in the future.^[55]^ These findings further strengthen the possible association of anemia with reduced myocardial oxygenation, myocardial fibrosis and adverse outcomes in the ESRD patients.

### 5.6. Limitations

This study should be considered as hypothesis generating only due to the smaller patient size. Although we were particularly careful during the T1 analysis in the apical slices of the left ventricle, artificially high T1 values might emerge with confounding variables due to the delineation of blood pool (partial volume effect) or adipose tissue in epicardium into ROIs. Moreover, the absence of specific interventricular septum T1 assessment and T2 mapping assessment is a potential confounder. Without histology verification, we cannot ensure that myocardial involvements observed in ESRD patients with anemia are representative of fibrosis. Finally, although we have found increased myocardial native T1 values in the ESRD patients with anemia, there was no discussion about the impact of specific treatment options on anemia, which require further exploration in the future.

## 6. Conclusions

We found elevated T1 values in ESRD population, implying diffuse myocardial fibrosis, could be quantitatively detected by using native T1 mapping without contrast. Furthermore, T1 values increased in ESRD patients with anemia, following independent correlations with anemia. In addition to discovering novel perspectives in the influence of anemia on myocardial fibrotic process, these findings may stimulate further research to investigate the significance of T1 value in promoting risk stratification and guiding cardiac interventions in ESRD patients.

## Acknowledgments

We appreciate those kind persons for their assistance in data analysis, language, and writing.

## Author contributions

**Conceptualization:** Lin Chen, Huayan Xu, Yi Zhang, Chunchao Xia, Li Rao, Yingkun Guo

**Data curation:** Lin Chen, Rong Xu, Huayan Xu, Yi Zhang, Zhenlin Li, Chunchao Xia

**Formal analysis:** Lin Chen, Rong Xu, Huayan Xu, Yi Zhang, Li Rao, Yingkun Guo

**Investigation:** Lin Chen, Zhigang Yang, Yingkun Guo

**Methodology:** Lin Chen, Rong Xu, Huayan Xu, Zhigang Yang, Yi Zhang, Zhenlin Li, Li Rao, Yingkun Guo

**Resources:** Lin Chen, Yi Zhang, Zhenlin Li, Chunchao Xia

**Software:** Lin Chen, Yi Zhang, Zhenlin Li, Chunchao Xia

**Visualization:** Lin Chen, Rong Xu, Zhenlin Li, Chunchao Xia, Yingkun Guo

**Writing – original draft:** Lin Chen

**Supervision:** Rong Xu, Huayan Xu, Zhigang Yang, Yi Zhang, Zhenlin Li, Yingkun Guo

**Validation:** Rong Xu, Huayan Xu, Zhigang Yang, Zhenlin Li, Yingkun Guo

**Writing – review & editing:** Rong Xu, Huayan Xu, Yingkun Guo

**Project administration:** Huayan Xu, Yingkun Guo

**Funding acquisition:** Zhigang Yang, Yingkun Guo
